# Comparison of Dementia Patients Admission Rates and Dementia Characteristics Before and During the COVID-19 Pandemic

**DOI:** 10.7759/cureus.19934

**Published:** 2021-11-27

**Authors:** Gulin Morkavuk, Ayca Demirkol, Gokalp Erdem Berber, Velanur Demirhan, Emine Simla Sahin, Pelin Akyuz, Alev Leventoglu

**Affiliations:** 1 Neurology, Ufuk University, Faculty of Medicine, Ankara, TUR; 2 Medicine, Ufuk University, Faculty of Medicine, Ankara, TUR

**Keywords:** admission rate, vulnerable groups, dementia, pandemic, covid-19

## Abstract

Background

The coronavirus disease 2019 (COVID-19) epidemic was recognized as a pandemic by the World Health Organization on March 2020. There have been significant changes in our lives due to the measures used to prevent the spread of the COVID-19 pandemic. Dementia patients are one of the most vulnerable groups who have difficulties in adapting to this situation. Our study aimed to compare the admission rate to the hospital and dementia characteristics of dementia patients in the COVID-19 pandemic and pre-pandemic periods.

Methods

Dementia patients admitted to the neurology outpatient clinic during the pandemic and pre-pandemic periods were included in the study. In these two periods, age, gender, dementia type, stage, age of onset, mini-mental state examination, reason for admission, vitamin B12, vitamin D, folic acid levels, brain imaging, electroencephalogram results were analysed retrospectively. Dementia characteristics and vitamin levels were compared.

Results

Two hundred and two dementia patients were included in the study. When the reasons for admission to the hospital were examined, the number of applications with the complaint of forgetfulness was highest in the pre-COVID period (53.1%); this rate was 37.8% in the COVID period. Also, 9.5% of patients were admitted for a drug prescription or medication report during the COVID period, while this rate was 1.6% in the pre-COVID period. Brain imaging was performed on 91 patients in the pre-COVID period, while 42 patients underwent imaging in the COVID period.

Conclusions

Although this study was performed with a limited population, it indicates that the COVID-19 pandemic indirectly affects the clinical conditions of people living with dementia.

## Introduction

Dementia is a progressive disease characterised by the impairment of more than one cognitive function due to central nervous system damage and the effects of daily living activities. Alzheimer’s disease (AD) accounts for 60%-70% of dementia patients. Vascular dementia (VD) is observed in 20%, Lewy body dementia (LBD) in 15% and frontotemporal dementia (FTD) in 5% [1,2]. In dementia, as the disease progresses, long-term memory impairment and language impairment develops gradually. Over time, disorientation starts, and after a certain period, patients become unable to perform their daily life activities alone and need help. Dementia patients find it difficult to adapt to changes and innovations in life due to memory problems.

The coronavirus disease 2019 (COVID-19) pandemic, where the first cases began to appear in China in December 2019, was declared a pandemic by the World Health Organization on March 11, 2020, the date of the first case in Turkey. There have been significant changes in our lives due to the measures to be taken to prevent the spread of the COVID-19 pandemic and the personal protective equipment that should be used. It is a fact that adults comply with this situation more quickly, but children and the elderly may have difficulty adapting. Dementia patients are one of the most vulnerable groups who have challenges in adapting to this situation. Although they have difficulty understanding what is happening in the environment and settling into new living conditions, it seems inevitable that they will deteriorate cognitively due to their isolation from the environment. The restrictions of individuals over 65 years of age, which are applied in our country as well as globally, may cause hospital admissions to be postponed or delayed in hospital admissions and make it difficult for the relatives of the elderly with forgetfulness to notice this situation. The prospective consequences of delayed diagnosis in dementia patients for whom the early diagnosis is critical is unpredictable. It is also unknown how long the pandemic will last, how these patients will be affected by the new isolation rules, and how their diseases will progress during the pandemic period. Based on all of this information, we aimed to examine the dementia patients admitted to our hospital during the COVID period and compare them with the dementia admissions of the previous year. The data obtained in this study aimed to raise awareness about the measures that should be taken for dementia patients to overcome the process more easily, the additional treatments that should be given, and perhaps the necessity of evaluating and monitoring dementia patients in every process with the telemedicine method.

## Materials and methods

Patient selection procedure

Dementia patients admitted to the Ufuk University Faculty of Medicine Neurology outpatient clinic with the complaint of amnesia, who were diagnosed with dementia, and who were followed up for dementia and admitted for control or other reasons were included in the study. We determined the COVID period to be between March 2020, when the cases started to appear in our country, and February 2021, and the pre-COVID period to be between March 2019 and February 2020 in order to include the same period as before COVID. During these two periods, the age, gender, dementia type, dementia stage, dementia onset age, mini-mental state examination (MMSE), the reason for application, comorbid diseases, vitamin B12, vitamin D, and folic acid levels were retrospectively obtained from the hospital data system, brain images from the patient files or hospital imaging system, and the electroencephalogram (EEG) results from the EEG laboratory archive. Dementia characteristics and vitamin levels of the two periods were compared. This study was performed according to the Declaration of Helsinki and with the approval of our hospital's Ethics Committee (Ethics Committee of Ufuk University Faculty of Medicine 2021-04-05).

Statistical analysis

Statistical analyses were performed using SPSS (Statistical Package for Social Sciences) version 22 software (IBM Corp., Armonk, NY). The compliance of quantitative variables to normal distribution was examined using visual (histogram) and analytical methods (Kolmogorov-Smirnov). Descriptive analyses were presented using median and minimum-maximum values for non-normally distributed variables. Pearson's chi-square and Fisher exact tests were used for nominal cross tables. Student’s T compared binary-scale parametric data and Mann-Whitney U test nonparametric data. A value of p < 0.05 was considered statistically significant.

## Results

Two hundred and two dementia patients were included in the study. There were 128 (53 male, 75 female) admissions in the pre-COVID period and 74 patients (22 male, 52 female) in the COVID period (Table 1).

**Table 1:** Gender distribution between groups.

		Gender		N	p-value
		Male	Female		
Group	Before the COVID-19 pandemic	53	75	128	
	During the COVID-19 pandemic	22	52	74	0.098
N		75	127	202	

When the reasons for admission to the hospital were examined, the number of applications with the complaint of forgetfulness was highest in the pre-COVID period with 53.1%. During the COVID period, the forgetfulness complaint constituted 37.8% of the admissions. In the pre-COVID period, the admission with the complaint of forgetfulness was statistically significantly higher than the COVID period (p = 0.009). Also, 9.5% of patients were admitted for a drug prescription or medication report during the COVID period, while this rate was 1.6% in the pre-COVID period. With these results, we can conclude that the low number of admissions with the complaint of amnesia during the COVID period, delayed admission to the hospital, or the amnesia of elderly patients living alone due to restrictions was not noticed by their relatives or was noticed late. Other admission reasons were follow-up admissions, headache, dizziness, behavioural changes, urinary incontinence, imbalances, hallucinations, walking difficulties, agitation, and confusion. As expected when evaluating dementia types, more AD was observed in both groups, and there was no statistically significant difference between the groups. The number of patients who were admitted to our outpatient clinic pre-COVID was more than during the COVID period. In correlation with this, the numbers of Parkinson's dementia and vascular dementia patients were also higher in the pre-COVID period. Creutzfeldt-Jakob disease (CJD) was seen in one patient in the pre-COVID period. Contrary to what was expected, while there was an admission for an LCD patient in the pre-COVID period, two patients were admitted during the COVID period (Table 2). When the groups were compared by the dementia stage at admission, no statistical difference was observed.

**Table 2:** Comparison of reason for admission, brain imaging, type of dementia and dementia stages between groups.

	Before the COVID-19 pandemic (n:128)	During the COVID-19 pandemic (n:74)	p-value
Reason for admission			
Forgetfulness	68	28	0.009
Follow-up admissions	43	33	
Behavioural changes	4	4	
Drug prescription or medication report	2	7	
Others	11	2	
Brain imaging			
MRI: atrophy, gliosis	50	33	0.033
MRI: chronic ischemia	10	2	
CT: atrophy	31	7	
Patients without brain imaging	37	32	
Type of dementia			
Alzheimer's dementia	112	67	0.594
Parkinson's dementia	7	2	
Vascular dementia	7	3	
Lewy body dementia	1	2	
Creutzfeldt–Jakob disease	1	0	
Dementia stages			
Early stage	76	40	0.362
İntermediate stage	45	26	
Advanced stage	7	8	

Brain imaging was performed on 91 patients in the pre-COVID period, 60 of which using magnetic resonance imaging (MRI), and 31 used computed tomography (CT). In the COVID period, only 42 patients, 35 MRI and 7 CT, underwent brain imaging (p = 0.033). We believe that the reason for the difference between the two groups was to try to shorten the hospitalisation period of the patients by performing as few examinations as possible during the COVID period (Table 2). There was no significant difference between the two groups in terms of age, vitamin D, B12, folate levels, EEG, MMSE, and dementia onset ages (Table 3).

**Table 3:** Comparison of age, age of onset of dementia, vitamin levels, EEG and MMSE results between groups. EEG: electroencephalogram; MMSE: mini-mental state examination.

	Before the COVID-19 pandemic	During the COVID-19 pandemic	p-value
Age, year, mean±SD, range	N:128	N:74	0.587
	78.53±7.4 (57-94)	77.92±8.2 (55-96)	
Dementia onset ages mean±SD, range	N: 61	N: 37	0.280
	76.6±8.4 (56-92)	74.7±9.1 (50-93)	
Vitamin D level, mean±SD, range	N: 56	N:29	0.677
	20.6±9.6 (3-44)	21.8±18.2 (3-97)	
Vitamin B12 level, mean±SD, median, range	N:101	N:49	0.137
	596±701, 416 (162-6000)	422±240, 354 (172-1227)	
Folate level mean±SD, median, range	N:86	N:47	0.321
	8±6.8, 6.3 (1.2-40)	7.9±4.1, 7.2 (2.1-20)	
EEG mean±SD, median, range	N:55	N:29	0.552
	8.4±1.1, 9 (5-11)	8.2±0.9, 9 (6-10)	
MMSE mean±SD, median, range	N:71	N:21	0.476
	21.9±3.3, 23 (15-28)	20.8±4.6, 23 (10-27)	

## Discussion

Today, it is known that the frequency of dementia increases day by day with the prolongation of life expectancy. Indeed, the World Health Organisation and the Alzheimer's Disease International 2016 Report identified this situation as a global public health priority [3]. Unfortunately, this vulnerable group is estimated to be one of the most affected patient groups in the COVID-19 pandemic. The COVID-19 pandemic exacerbates the vulnerability of elderly patients with cognitive impairment, particularly those who depend on family or caregivers for their daily care. It becomes a predictable fact that dementia patients with increased confusion due to restrictions, new practices, and lack of communication may deteriorate cognitively in this process.

There are studies considering that measures taken to slow the spread of the virus may contribute to the worsening of loneliness, behavioural symptoms, and cognitive functions in dementia patients. One of them is a questionnaire applied by phone to 139 dementia patients and their caregivers after the first 30 days of quarantine in Italy. In this survey study, the perceived changes in the clinical status of dementia patients and their caregivers in the last 30 days were evaluated. It was reported that cognitive symptoms, especially memory and orientation abilities, worsen in one-third of the patients, and behavioural disorders with agitation/aggression, apathy, and depression were reported to worsen in half of the patients. A decrease in personal care and daily living activities was reported in 19 patients. The clinical changes observed in some of the patients necessitated new adjustments in ongoing pharmacological therapy or the introduction of new therapies. In the same study, half of the caregivers reported higher stress levels and fatigue compared to the previous month [4]. There are other studies all over the world showing cognitive, neuropsychiatric, and functional deterioration in dementia patients during the restraint periods [5]. In a study in which 40 patients with mild AD and mild cognitive impairment (MCI) who participated in a cognitive-stimulating program in Spain were evaluated, it was reported that the neuropsychiatric symptoms of the patients worsened after five weeks of quarantine [6]. In another study conducted with frontotemporal dementia patients, it was observed that after quarantine, 53% of the patients had worsening cognitive functions, behaviour, and language functions, especially in memory compared to six months ago [7]. In our study, in the pre-pandemic period, 59.3% of the patients were early stage, 35.1% were middle stage, and 5.4% were advanced stage dementia patients; in contrast, during the pandemic period, 54% were early-stage dementia patients, 35.1% were middle stage, and 10.8% were advanced stage dementia patients. Although there was no statistically significant difference, the rate of advanced stage dementia observed was higher in COVID-period dementia admissions. We think that this finding may be an indication that dementia progressed faster during the pandemic period. However, we believe that prospective studies on this subject will yield more accurate results.

Another concern is the impact of the pandemic on people in the pre-clinical stage of dementia or experiencing mild cognitive changes. In a study conducted in September 2020 and published in 2021, 36 dementia and MCI patients who had MMSE before the restrictions were re-evaluated by MMSE after the restrictions. According to the results, disease severity and stage changed in 11 patients during the study. One MCI developed into mild dementia, six mild dementia progressed to the medium stage, and four moderate dementia developed to severe dementia. While the average MMSE was 17.3 pre-lockdown, it was 13.9 post-lockdown. It was observed that the most affected cognitive area was memory. As a result, in this study, it was concluded that cognitive functions decreased more rapidly during quarantine in patients with dementia and MCI compared to the pre-pandemic period [8]. In another study published in 2021, caregivers of MCI and dementia patients were evaluated with a questionnaire. Two hundred and four caregivers, mostly women, were included in the study. The most affected areas were determined to be communication, mood, movement, and adaptation to new measures; it was observed that the impact in the middle/advanced dementia patients was more pronounced than in the MCI and mild dementia groups. Also, caregiver burden was higher in the middle/advanced dementia group, as expected [9]. When the reasons for admission were examined in our study, the admission rate with the complaint of forgetfulness was 53.1% in the pre-pandemic group, while it was 37.8% during the pandemic period. We can conclude that the low number of admissions with the complaint of amnesia during the COVID period, delayed admission to the hospital, or the amnesia of elderly patients living alone due to restrictions was not noticed by their relatives or was noticed late. These results suggest that the anxiety felt by people in the pre-clinical stage of dementia or those with mild cognitive changes is not unwarranted.

Chronic diseases such as dementia are of particular concern, not only because they are associated with higher hospitalisation and mortality rates but also because COVID-19 worsens the vulnerability of those with cognitive impairment. The physical distance rule recommended during the epidemic has turned into social isolation by increasing measures due to an increase in the number of cases. Anxiety, depression, and the decrease in external stimuli due to social isolation cause a deterioration in cognitive functions or the progression of the existing disorder. This situation has led people to communicate on the phone or make video calls on the internet [10].

Telemedicine is defined as a tool for providing remote healthcare services with the use of telecommunication technology [11]. Social distancing can significantly compromise the quality of life and long-term health of older adults living in the community and their caregivers. Telemedicine appears to be an open and cost-effective alternative to providing this vulnerable group with social distancing measures during the ongoing COVID-19 pandemic [12]. Therefore, compared to traditional phone calls alone, video conferencing can increase social interaction in older adults when the opportunity for face-to-face contact is restricted by social distance. It is thought that telehealth through video conferencing can minimise the potential adverse effects of social distancing measures required by the COVID-19 pandemic. In 2020, a study was organised in Hong Kong by separating a group with neurocognitive disease and caregivers, 30 patient and caregiver pairs as the intervention group, and 30 patient and caregiver pairs as a control group. The communication was provided by only weekly phone calls in the control group. In the intervention group, an additional video communication was provided with a 30-minute video conference method. The cognitive evaluation of the patients was performed using the Montreal Cognitive Assessment (MoCA), the quality of life of the caregivers with Short Form-36 (SF-36), and the caregiver burden with The Zarit Burden Interview Scale (ZBI) pre-test and post-test. It was found that there was a deterioration in MoCA, ZBI, and SF-36 only in the group who was interviewed by phone. It was found that there was no deterioration in the tests in the group that was interviewed weekly with the video conference method. Varying degrees of improvements in physical and mental health (SF-36) and perceived burden (ZBI) were observed among caregivers in the videoconferencing group [13]. Studies indicate that telemedicine has a high level of satisfaction and effectiveness at a low cost and is very convenient and easily accessible [14]. It was also reported that it provides clinical results equivalent to face-to-face services [11,15]. Telemedicine not only replaces face-to-face appointments, but includes the possibility of improving mental health, new ways to deliver care, more frequent but shorter encounters, and early intervention opportunities [14,16]. Telemedicine can help to reduce the risk of developing adverse mental health outcomes associated with reduced social contact and less access to healthcare. Also, it allows for real-time, remote health support, allowing adequate medication adjustments when needed without exposing the patient and caregiver to infection risks. Telemedicine seems to continue to have an essential role in evaluating patients with cognitive impairment even after the COVID-19 pandemic is under control.

For dementia patients to pass through the pandemic process without cognitive impairment, health professionals and their relatives have a great responsibility. In this regard, the patient's relative should be informed in detail by the doctor. Especially in this period, activities that the patient can do at home or in a virtual environment (such as board games, newspaper reading, puzzle solving) should be planned for them to have an enjoyable and productive time. They can also assist with supervised housework (such as cooking, setting the dinner table). These activities stimulate them and build their self-esteem as they feel useful. Tasks such as pursuing hobbies, and caring for a pet or plant will also be helpful if possible. For the patient not to feel lonely, they should be relieved by making them feel that they are going out. Technologies such as computers, tablets, or smartphones can be used to keep patients in touch with their loved ones and friends. If possible, a fixed life order should be presented in their environment without disturbing the daily routine. Short walks in the garden or at home, passive exercises can help to prevent behavioural disorders. The patient may have difficulty understanding the information s/he hears about the epidemic on television and may be confused. In this case, brief and straightforward explanations can be made. Instead of explaining details, it would be helpful to explain the main lines in a simple language that they can understand. Also, reminders should be made when necessary for hygiene practices. The use of masks should also be reminded and patients should be shown how to wear them when necessary. Dementia patients may have difficulty perceiving the risks associated with COVID-19, which can cause discomfort, agitation, and sleep disturbances. Finally, it is crucial to remember that the caregiver will be exposed to stress in many more situations than usual and must take care of their own health. Therefore, more than ever, alternative ways should be found for the caregivers to relax since they have small spaces for themselves and seek strategies to manage anxiety [17].

## Conclusions

Our study shows how much the rate of admission to the hospital and the examinations performed in patients with dementia, one of the most vulnerable groups during the COVID-19 period, have decreased due to restrictions. This situation will cause delay in the diagnosis of patients with early stage dementia and MCI and will prevent adequate treatment. We believe that it is a very important detail that dementia patients should always be followed up under all circumstances. Dementia patients and their caregivers can be offered alternative support options, such as home healthcare services, by telemedicine methods, or by taking necessary precautions by experienced health professionals. These applications will allow us to monitor the clinical condition of patients and to identify emergencies promptly that we should prioritise. In addition, thanks to the continuity of patient follow-up, it will be possible to detect cognitive and behavioral problems in patients, and necessary treatments will be started on time.

## References

[1] McKeith IG (2002). Dementia with Lewy bodies. Br J Psychiatry.

[2] Arvanitakis Z (2010). Update on frontotemporal dementia. Neurologist.

[3] (2021). World Alzheimer Report 2016. Improving healthcare for people living with dementia. https://www.alzint.org/u/WorldAlzheimerReport2016.pdf.

[4] Canevelli M, Valletta M, Toccaceli Blasi M (2020). Facing dementia during the COVID-19 outbreak. J Am Geriatr Soc.

[5] Alonso-Lana S, Marquié M, Ruiz A, Boada M (2020). Cognitive and neuropsychiatric manifestations of COVID-19 and effects on elderly individuals with dementia. Front Aging Neurosci.

[6] Lara B, Carnes A, Dakterzada F, Benitez I, Piñol-Ripoll G (2020). Neuropsychiatric symptoms and quality of life in Spanish patients with Alzheimer's disease during the COVID-19 lockdown. Eur J Neurol.

[7] Capozzo R, Zoccolella S, Frisullo ME (2020). Telemedicine for delivery of care in frontotemporal lobar degeneration during COVID-19 pandemic: results from Southern Italy. J Alzheimers Dis.

[8] Ismail II, Kamel WA, Al-Hashel JY (2021). Association of COVID-19 pandemic and rate of cognitive decline in patients with dementia and mild cognitive impairment: a cross-sectional study. Gerontol Geriatr Med.

[9] Tsapanou A, Papatriantafyllou JD, Yiannopoulou K (2021). The impact of COVID-19 pandemic on people with mild cognitive impairment/dementia and on their caregivers. Int J Geriatr Psychiatry.

[10] Altın Z (2020). Elderly people in Covid-19 outbreak. Tepecik Eğit. ve Araşt. Hast. Dergisi.

[11] Gentry MT, Lapid MI, Rummans TA (2019). Geriatric telepsychiatry: systematic review and policy considerations. Am J Geriatr Psychiatry.

[12] Banbury A, Nancarrow S, Dart J, Gray L, Dodson S, Osborne R, Parkinson L (2020). Adding value to remote monitoring: co-design of a health literacy intervention for older people with chronic disease delivered by telehealth - The telehealth literacy project. Patient Educ Couns.

[13] Lai FH, Yan EW, Yu KK, Tsui WS, Chan DT, Yee BK (2020). The protective impact of telemedicine on persons with dementia and their caregivers during the COVID-19 pandemic. Am J Geriatr Psychiatry.

[14] Donelan K, Barreto EA, Sossong S (2019). Patient and clinician experiences with telehealth for patient follow-up care. Am J Manag Care.

[15] Kim H, Jhoo JH, Jang JW (2017). The effect of telemedicine on cognitive decline in patients with dementia. J Telemed Telecare.

[16] Soares WB, Silvestre IT, Lima AM, de Almondes KM (2020). The influence of telemedicine care on the management of behavioral and psychological symptoms in dementia (BPSD) risk factors induced or exacerbated during the COVID-19 pandemic. Front Psychiatry.

[17] Altable M, Moisés de la Serna J (2020). Caregivers and Alzheimer disease in the COVID-19 pandemic. J Glob Health.

